# Mitochondrial dysfunction in adult midbrain dopamine neurons triggers an early immune response

**DOI:** 10.1371/journal.pgen.1009822

**Published:** 2021-09-27

**Authors:** Roberta Filograna, Seungmin Lee, Katarína Tiklová, Mara Mennuni, Viktor Jonsson, Markus Ringnér, Linda Gillberg, Elena Sopova, Oleg Shupliakov, Camilla Koolmeister, Lars Olson, Thomas Perlmann, Nils-Göran Larsson

**Affiliations:** 1 Department of Medical Biochemistry and Biophysics, Karolinska Institutet, Stockholm, Sweden; 2 Department of Cell and Molecular Biology, Karolinska Institutet, Stockholm, Sweden; 3 Department of Biology and Biological Engineering, National Bioinformatics Infrastructure Sweden, Science for Life Laboratory, Chalmers University of Technology Gothenburg, Sweden; 4 Department of Biology, National Bioinformatics Infrastructure Sweden, Science for Life Laboratory, Lund University, Lund, Sweden; 5 Department of Neuroscience, Karolinska Institutet, Stockholm, Sweden; 6 Institute of Translational Biomedicine, St Petersburg State University, St Petersburg, Russia; University of Cologne, GERMANY

## Abstract

Dopamine (DA) neurons of the midbrain are at risk to become affected by mitochondrial damage over time and mitochondrial defects have been frequently reported in Parkinson’s disease (PD) patients. However, the causal contribution of adult-onset mitochondrial dysfunction to PD remains uncertain. Here, we developed a mouse model lacking Mitofusin 2 (MFN2), a key regulator of mitochondrial network homeostasis, in adult midbrain DA neurons. The knockout mice develop severe and progressive DA neuron-specific mitochondrial dysfunction resulting in neurodegeneration and parkinsonism. To gain further insights into pathophysiological events, we performed transcriptomic analyses of isolated DA neurons and found that mitochondrial dysfunction triggers an early onset immune response, which precedes mitochondrial swelling, mtDNA depletion, respiratory chain deficiency and cell death. Our experiments show that the immune response is an early pathological event when mitochondrial dysfunction is induced in adult midbrain DA neurons and that neuronal death may be promoted non-cell autonomously by the cross-talk and activation of surrounding glial cells.

## Author summary

Parkinson’s disease (PD) is a common neurodegenerative disorder characterized by progressive loss of dopamine (DA)-producing neurons and strongly compromised motor performance. Multiple observations suggest that DA neurons are particularly prone to acquire mitochondrial damage in adult life. This acquired mitochondrial dysfunction likely impairs DA neuron function and contributes to cell death. To study the consequences of adult-onset mitochondrial dysfunction in DA neurons, we generated a conditional activatable knockout mouse model lacking *Mitofusin 2*, *a* key regulator of mitochondrial homeostasis. This animal model allows the induction of mitochondrial dysfunction selectively in adult DA neurons and leads to motor defects and the typical pattern of neurodegeneration seen in PD. By studying gene expression in isolated DA neurons at early disease stages and by using *in situ* approaches on brain sections, we report an early onset of an inflammatory response. Inflammation is present already when the mutant DA neurons display the first signs of mitochondrial fragmentation and precedes the onset of respiratory chain dysfunction and neurodegeneration. The inflammatory response in DA neurons and activation of surrounding glia thus likely exacerbates or drives the neurodegenerative process in this animal model of adult-onset PD.

## Introduction

Most neuronal cells have a life span similar to that of the whole organism and are rarely or never replaced [1]. As a consequence, neurons are prone to accumulate defects which affect their function and plasticity, and even compromise their long-term survival. The cortical surface of the cerebellum and certain brain nuclei, e.g. *Substantia nigra pars compacta* (*SNpc*), are particularly vulnerable to acquired damage [2,3], whereas other regions, e.g. hippocampus, putamen, and hypothalamus almost completely preserve their neuronal integrity during adult life [4]. The loss of dopamine (DA) neurons in *SNpc* occurs at an estimated rate of ~5–10% per decade [5,6]. Notably, a massive degeneration of this neuronal population accounts for the motor symptoms found in Parkinson’s disease (PD) patients. The selective vulnerability of DA neurons seems to be caused by their intrinsic biochemical and physiological properties. DA neurons in *SNpc* have rhythmic electrical (pacemaker) activity and experience increased oxidative stress, presumably due to the high dopamine synthesis rate [7]. The *SN* is also highly enriched in microglia cells [8], which, if activated, may generate a potentially detrimental pro-inflammatory environment. In addition, DA neurons are thought to be particularly sensitive to mitochondrial damage, which is mainly acquired during the lifespan of the neuron rather than inherited. In fact, somatic deletions in the mitochondrial DNA (mtDNA) accumulate in DA neurons in *SN* of aged humans [9] and PD patients [10,11] and lead to a mosaic pattern of respiratory chain deficiency.

Over the last decades, the role of mitochondrial dysfunction in the pathophysiology of PD has been much debated (reviewed in [12]). Although mitochondrial impairment is heavily implicated in both idiopathic and familial forms of PD, the precise contribution of these organelles to neurodegeneration remains unclear. There is experimental evidence that mitochondria are required to maintain specific cellular functions in DA neurons, such as anterograde axonal transport [13] and DA release by nerve terminals in the striatum [14]. In fact, mouse models with deletions [15] or depletion of mtDNA [16] selectively in midbrain DA neurons mirror the motor phenotypes and the typical neurodegeneration present in PD patients. One weakness with these sets of experiments is that the mitochondrial defects are induced in neurons already during the embryonic stage, which argue that the observed Parkinson-like phenotypes can be the result of both neurodevelopmental and neurodegenerative processes. To study the effects of adult-onset mitochondrial damage in PD, we disrupted the *Mitofusin 2 (Mfn2)* gene in midbrain DA neurons of adult mice. The *Mfn2* gene encodes a key component of mitochondrial fusion machinery and is therefore a major player in several mitochondrial pathways, e.g. trafficking, turnover, contacts with other organelles and organelle homeostasis (reviewed in [17,18]). In mice, the tissue-specific ablation of *Mfn2* in different neuronal circuits causes abnormalities in mitochondrial morphology and severe neurological defects [19,20]. Here, we identify a detailed timeline of molecular events driving the severe and progressive parkinsonism in mice with disruption of *Mfn2* in the adult nigrostriatal DA system. By using transcriptomic analyses of isolated adult midbrain DA neurons, we show that loss of mitochondrial homeostasis triggers an early-onset immune response, that precedes DA neuron death and therefore likely drives or exacerbates the degenerative process.

## Results and discussion

### Mice with adult-onset degeneration of midbrain DA neurons

**Fig 1:**
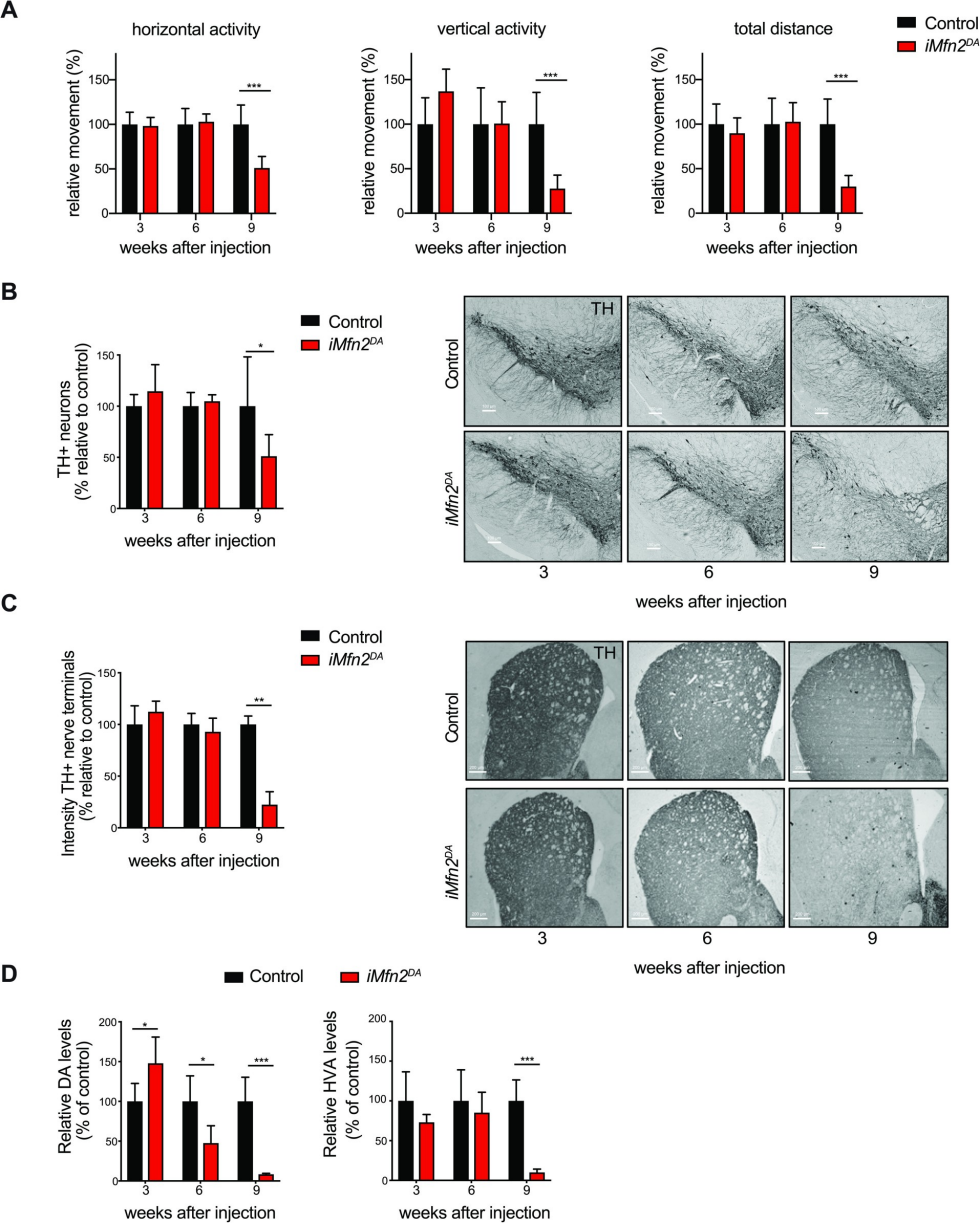
Tamoxifen-injected *iMfn2*^DA^ mice show impaired locomotion and DA neurodegeneration. Control and *iMfn2*^DA^ mice were analyzed at 3, 6 and 9 weeks after tamoxifen or vehicle injection. (A) Spontaneous motor activity (horizontal activity, vertical activity and total distance) was measured in open field. ***p< 0.001, n>14. (B-C) Representative images of TH-like immunoreactivity in section from midbrain (Scale bars: 100 μm) and striatum (Scale bars: 200 μm), right panels. Quantification of TH-positive DA neurons in the midbrain and TH-immunoreactive nerve terminals in the striatum, left panels. ***p< 0.001 n = 3. (D) Analysis of DA and HVA levels in the striatum. Data are shown as mean ± SD. ***p<0.001 n≥5.

To generate mice with mitochondrial dysfunction in adult DA neurons, we performed crosses to obtain *iMfn2*^DA^ mice that are homozygous for a loxP-flanked *Mfn2* allele and heterozygous for an allele expressing tamoxifen-inducible Cre-recombinase [21] under control of the DA transporter (DAT, *Slc6a3*) promoter (genotype: *Mfn2 loxP/loxP; +/Dat-creERT2*). To activate Cre-mediated recombination, *iMfn2*^DA^ mice were injected intraperitoneally with tamoxifen for five consecutive days at the age of 5–7 weeks (S1A Fig). The resulting mice showed a very profound decrease in MFN2, both at transcript and protein levels, at 3 weeks after tamoxifen injection (S1B and S1C Fig). As consequence, mice manifested a drastic reduction of life span with maximal longevity of 12 weeks after injection (S1D Fig) and a significant decline in body weight at 10 weeks after injection (S1E Fig). When tested in an open-field setup, tamoxifen-injected *iMfn2*^DA^ mice manifested decrease of horizontal activity, vertical activity (rearing) and total locomotion distance at 9 weeks after tamoxifen injection (Fig 1A), whereas motor abilities were unchanged at 3 and 6 weeks (Fig 1A). The decreased locomotion had a distinct neuroanatomical basis as histology of brains from tamoxifen-injected *iMfn2*^DA^ mice showed degeneration of the midbrain nigrostriatal DA system (Fig 1B and 1C). Quantification of tyrosine hydroxylase (TH) expression identified ~50% reduction in positive nerve cell bodies in *SN* (Fig 1B) and ~80% reduction in the striatal DA innervation (Fig 1C) in *iMfn2*^DA^ mice analyzed at 9 weeks after tamoxifen injection. There were no significant differences between knockouts and controls at earlier time points (Fig 1B and 1C). The profound degeneration of the midbrain DA system in tamoxifen-treated *iMfn2*^DA^ mice was further substantiated by measurements of levels of DA and its metabolite homovanillic acid (HVA) in striatal homogenates by using ultraperformance liquid chromatography-tandem mass spectrometry (UPLC-MS/MS). The DA levels were slightly increased at 3 weeks after tamoxifen injection, while there was a significant DA depletion at 6 weeks which became more profound at 9 weeks (Fig 1D). The levels of HVA were significantly affected only at the late-disease stage (Fig 1D), whereas the levels of serotonin (5-hydroxytryptamine, 5-HT) in the striatum were unchanged over time (S1F Fig). In summary, these findings show that tamoxifen-injected *iMfn2*^DA^ mice exhibit a severe parkinsonism in adulthood caused by degeneration of the midbrain DA system.

### Mitochondrial dysfunction in adult midbrain DA neurons

**Fig 2:**
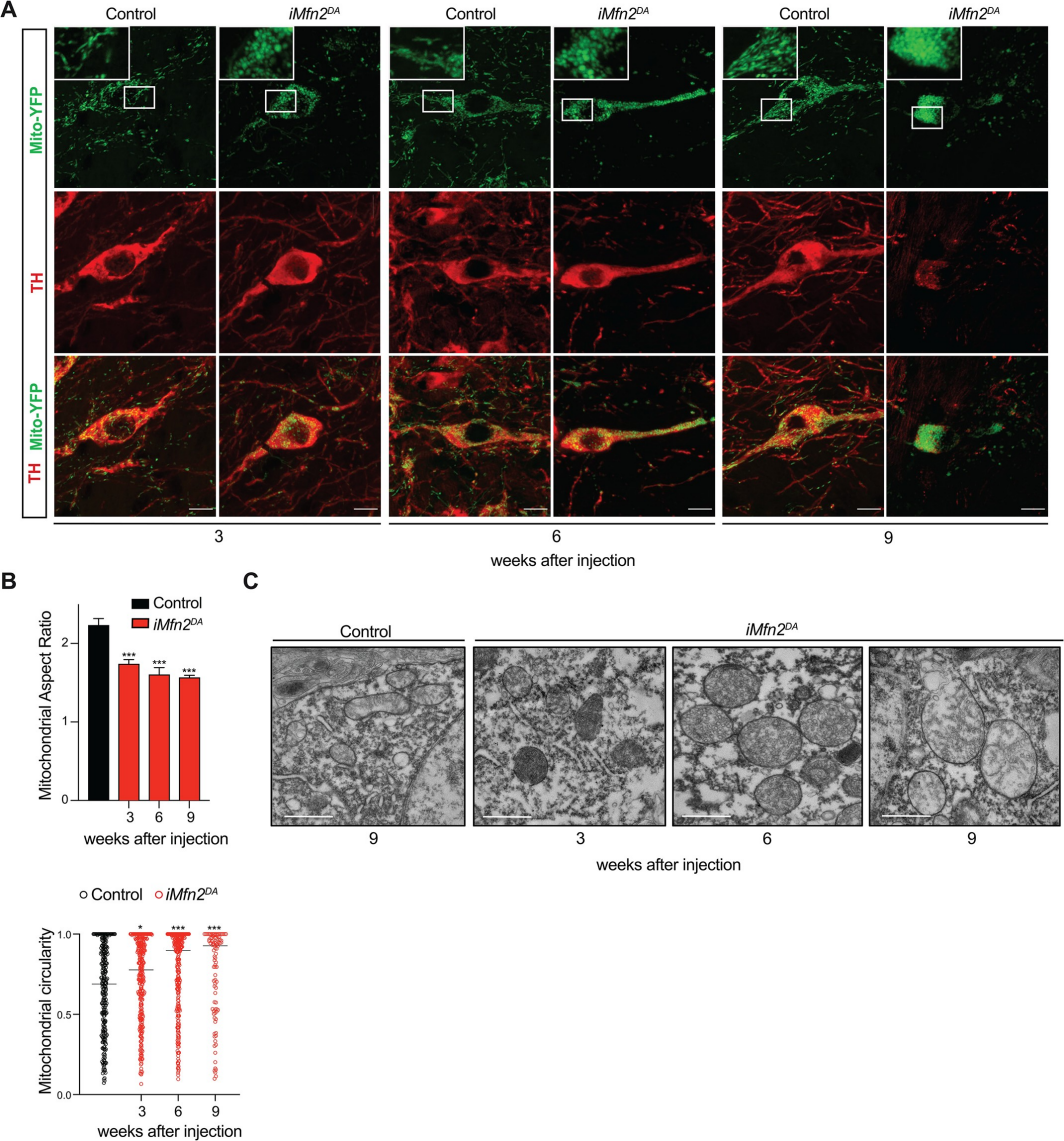
Loss of *Mfn2* in adult DA neurons affects mitochondrial morphology and cristae structure. Analyses were performed 3, 6, and 9 weeks after tamoxifen injection in control and *iMfn2*^DA^ mice. (A) Representative confocal microscopy images of YFP-labelled mitochondria (green) in TH immunoreactive neurons (red) (Scale bars: 10 μm). (B) Quantification of aspect ratio and mitochondrial circularity from confocal images in TH+ DA neurons. AR data are shown as mean ± SD and circularity data as median of individual mitochondria. ***p< 0.00, n = 3 for each genotype. (C) Representative electron microscopy images of mitochondrial cristae structure (Scale bars: 1 μm).

To assess mitochondrial morphology in the degenerating midbrain DA neurons, we introduced an allele that induces expression of mitochondrially targeted YFP (mito-YFP) after Cre-recombinase excision of a STOP-sequence [13]. Fluorescently labelled mitochondria in TH-expressing neurons in the midbrain (S2A Fig) showed a gradually compromised integrity of the mitochondrial network in tamoxifen-injected *iMfn2*^DA^ mice (Fig 2A). In the perinuclear region of the soma, mitochondria became highly fragmented already 2–3 weeks after tamoxifen injection and progressively more rounded and swollen after 6–9 weeks (Figs 2A and S2B), as demonstrated by decreased mitochondrial aspect ratio and increased mitochondrial circularity (Fig 2B). Electron microscopy (EM) analysis of single organelles confirmed the initial mitochondrial fragmentation and the consequent enlargement (S2C–S2E Fig), and also revealed structural abnormalities in mitochondrial cristae structure at 6 and 9 weeks after injection (Fig 2C). At 6 weeks, a disruption of the outer mitochondrial membrane (OMM) was detected in single mitochondrial profiles, and at 9 weeks, about 10% of the mitochondria in the perinuclear region displayed ruptured OMM (S2F Fig), likely due to the alterations of mitochondrial network and the consequent osmotic swelling of the mitochondrial matrix.

**Fig 3:**
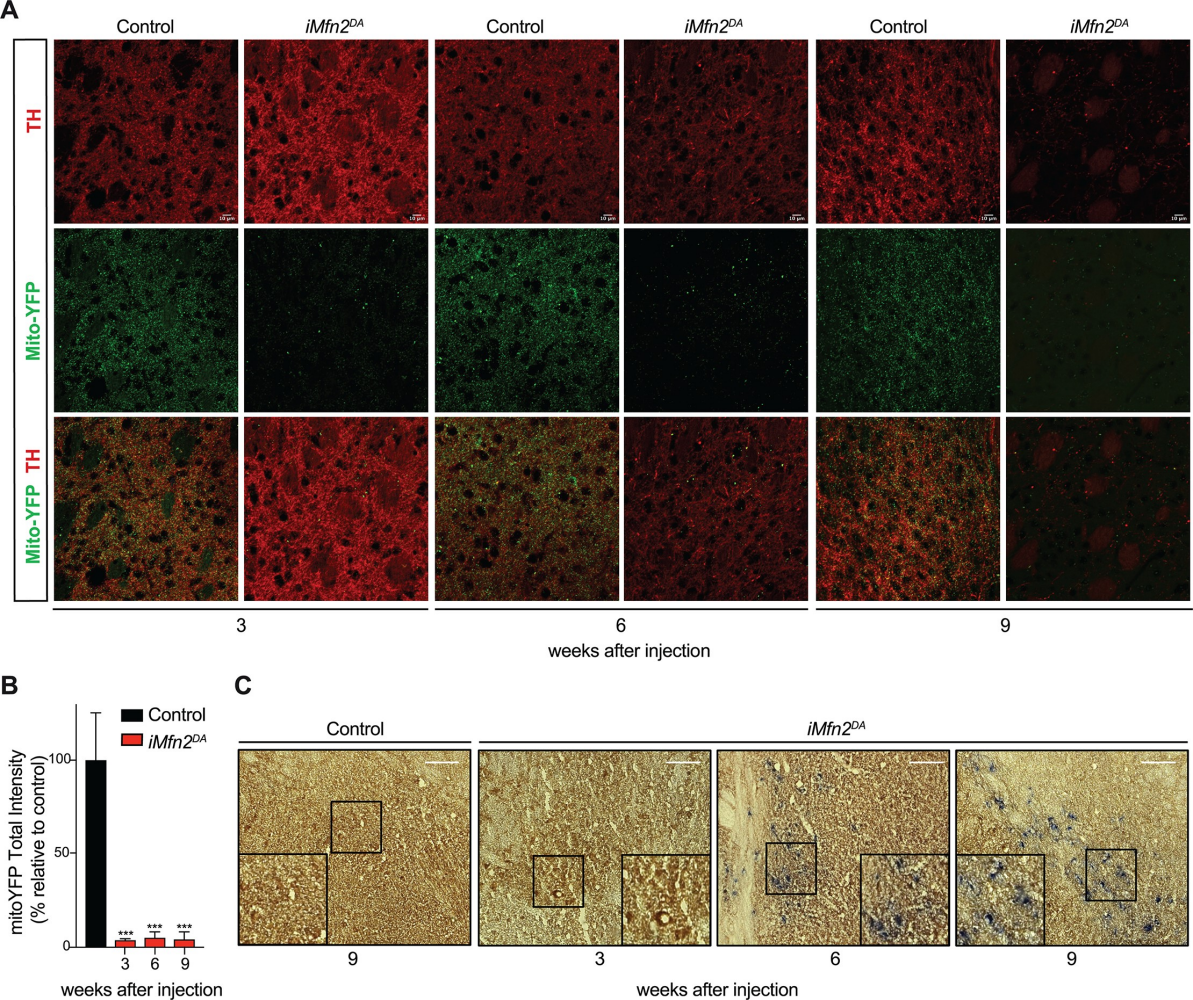
Tamoxifen-injected *iMfn2*^DA^ mice exhibit impaired axonal mitochondrial transport and OXPHOS deficiency. (A) MitoYFP-labelled mitochondria in TH+ nerve terminals of the striatum at 3, 6 and 9 weeks after tamoxifen injection (Scale bars: 10 μm). (B) Quantificantion of mitoYFP-labelled mitochondria total intensity in the striatum of control and tamoxifen-injected *iMfn2*^*DA*^ mice at 3, 6 and 9 weeks after tamoxifen injection. Data are shown as mean ± SD. *p< 0.05, ***p< 0.001, n = 3 for genotype. (C) Cytochrome *c* oxidase and succinate dehydrogenase (COX/SDH) double-labelling enzyme histochemistry of the midbrain (Scale bars: 100 μm).

The analysis of the mitochondrial distal pool in TH positive nerve terminals identified a dramatic decrease (~95%) in the amount of mito-YFP labelled mitochondria already at 3 and 6 weeks after injection (Fig 3A and 3B). At 9 weeks, the severe depletion of striatal mitochondria corresponded to a massive reduction in TH immunoreactive DA fibers in *iMfn2*^*DA*^ mice (Figs 1C and 3A). Notably, the few mitochondria found in axonal terminals (~5%) preserved their morphology and cristae organization even at 6 and 9 weeks after injection (S2G Fig). These data therefore confirm that the fragmented mitochondria fail to be transported in the DA axons, as we and others have previously reported [20,22], suggesting that the maintenance of the mitochondrial integrity is a requirement for axonal mitochondrial transport. Furthermore, our results indicate that in tamoxifen-injected *iMfn2*^*DA*^ mice the degeneration of the nigrostriatal system occurs in a retrograde fashion, i.e. from the terminal towards the cell body.

To assess oxidative phosphorylation (OXPHOS) function, we analyzed the activities of cytochrome c oxidase (COX) and succinate dehydrogenase (SDH) by a combined enzyme histochemical staining of midbrain tissues. At 3 weeks after tamoxifen injection, all midbrain DA neurons of *iMfn2*^DA^ mice appeared brown consistent with preserved COX activity. In contrast, at 6 and 9 weeks after injection, a substantial proportion of midbrain cells appeared blue, consistent with a profound decline in COX activity (Fig 3C).

Taken together, these findings show that tamoxifen-injected *iMfn2*^DA^ mice develop a severe adult-onset mitochondrial dysfunction in midbrain DA neurons, manifested as a progressive fragmentation of the mitochondrial network, swelling of individual mitochondria, abnormal mitochondrial ultrastructure and deficient respiratory chain function.

### Impaired mitochondrial homeostasis causes mtDNA depletion and an early immune response

**Fig 4:**
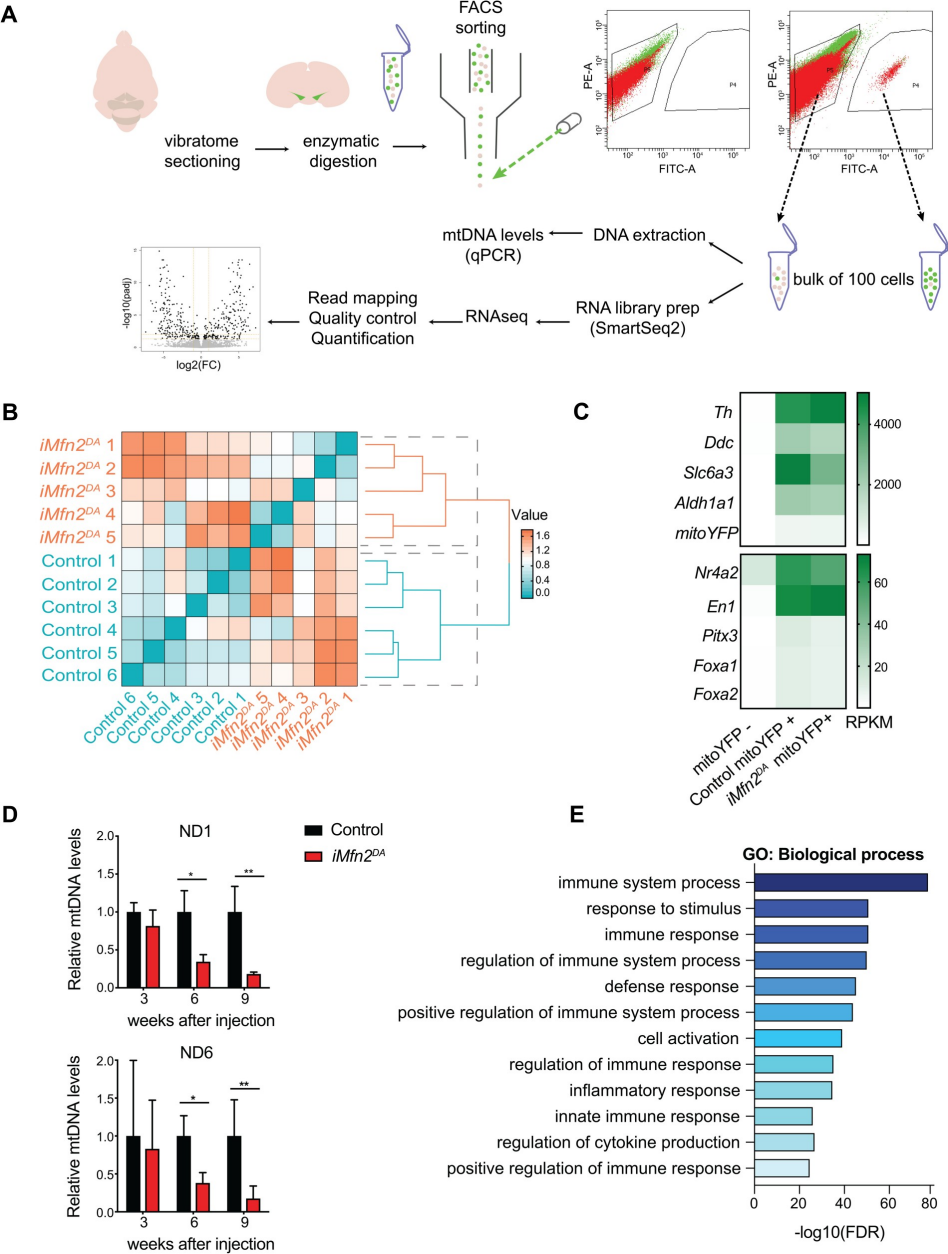
Immune response and mtDNA depletion in tamoxifen-injected *iMfn2*^DA^ mouse brains. (A) Experimental workflow: vibratome sections were enzymatically digested and mechanically triturated resulting in a single cell suspension; the bulk of mitoYFP positive (mitoYFP+) cells and mitoYFP negative (mitoYFP-) cells were collected by FACS; samples were used for either mtDNA quantification or for RNA library preparation followed by RNAseq. (B) Hierarchical clustering analysis of RNAseq data from DA neurons isolated from n = 5 *iMfn2*^DA^ and n = 6 control mice. (C) Heatmap showing that RNA levels (Reads Per Kilobase Million, RPKM) of genes encoding DA neuron markers (*Th*, *Ddc*, *Scl6a3*, and *Aldh1a1*) and transcription factors (*Nr4a2*, *En1*, *Pitx3*, *Foxa1* and *Foxa2*) are highly enriched in mitoYFP+ samples. Data are shown as mean of the RPKM in control (n = 6), *iMfn2*^DA^ (n = 5), and mitoYFP- (n = 3) mice. (D) Relative mtDNA levels (ND1/18S rRNA and ND6/18S rRNA) measured in FACS-sorted DA neurons. *p< 0.05, **p<0.01, n = 4. Data are shown as mean ± SD. (E) Gene ontology (GO) analysis showing the most dysregulated biological processes in FACS-sorted DA neurons isolated from *iMfn2*^DA^ mice at 3 weeks after tamoxifen injection.

To study pathophysiological events, we performed analysis of isolated adult DA neurons from tamoxifen-induced *iMfn2*^DA^ and control mice. We used a protocol for isolation and enrichment of midbrain DA neurons from mito-YFP mouse brains based on enzymatic tissue dissociation and FACS-sorting (Fig 4A). The expression of mito-YFP in isolated cells was independently validated by confocal microscopy (S3A Fig). We collected mito-YFP positive and mito-YFP negative cells from control and knockout mice and prepared libraries for RNAseq analysis using the Smart-seq2 protocol [23] (Fig 4A). Sequences were mapped to a total of 39468 genes of which 23217 protein-coding genes were used for the downstream analyses. Hierarchical clustering analysis was performed using the most variably expressed genes and two distinct groups were distinguished corresponding to the genotypes (Fig 4B). Well-validated DA neuronal markers (e.g. *Th*, *Ddc* and *Slc6a3*) and transcription factors (e.g. *Nr4a2*, *En1*) were found abundantly expressed in mito-YFP positive cells from controls and knockouts and were almost undetectable in mito-YFP negative populations (Fig 4C). After confirming that the mito-YFP positive samples were highly enriched with DA neurons, FACS-sorted cells were used to measure mtDNA levels by qPCR. Notably, at 3 weeks after tamoxifen injection, the mtDNA copy number was unaffected (Fig 4D), although the mitochondrial network was highly fragmented (Fig 2A). In contrast, mtDNA levels were decreased to 30–40% at 6 weeks and to 18% at 9 weeks in DA neurons isolated from tamoxifen-injected *iMfn2*^DA^ mice when compared with controls (Fig 4D). We therefore conclude that perturbations of the mitochondrial network precede mtDNA depletion, arguing that the loss of mitochondrial homeostasis is the main cause of the observed defect. These findings are consistent with a previous report from us showing that cardiomyocytes lacking mitochondrial fusion develop a reduction in mtDNA copy number due to an imbalanced stoichiometry of the mtDNA replisome protein components [24].

**Fig 5:**
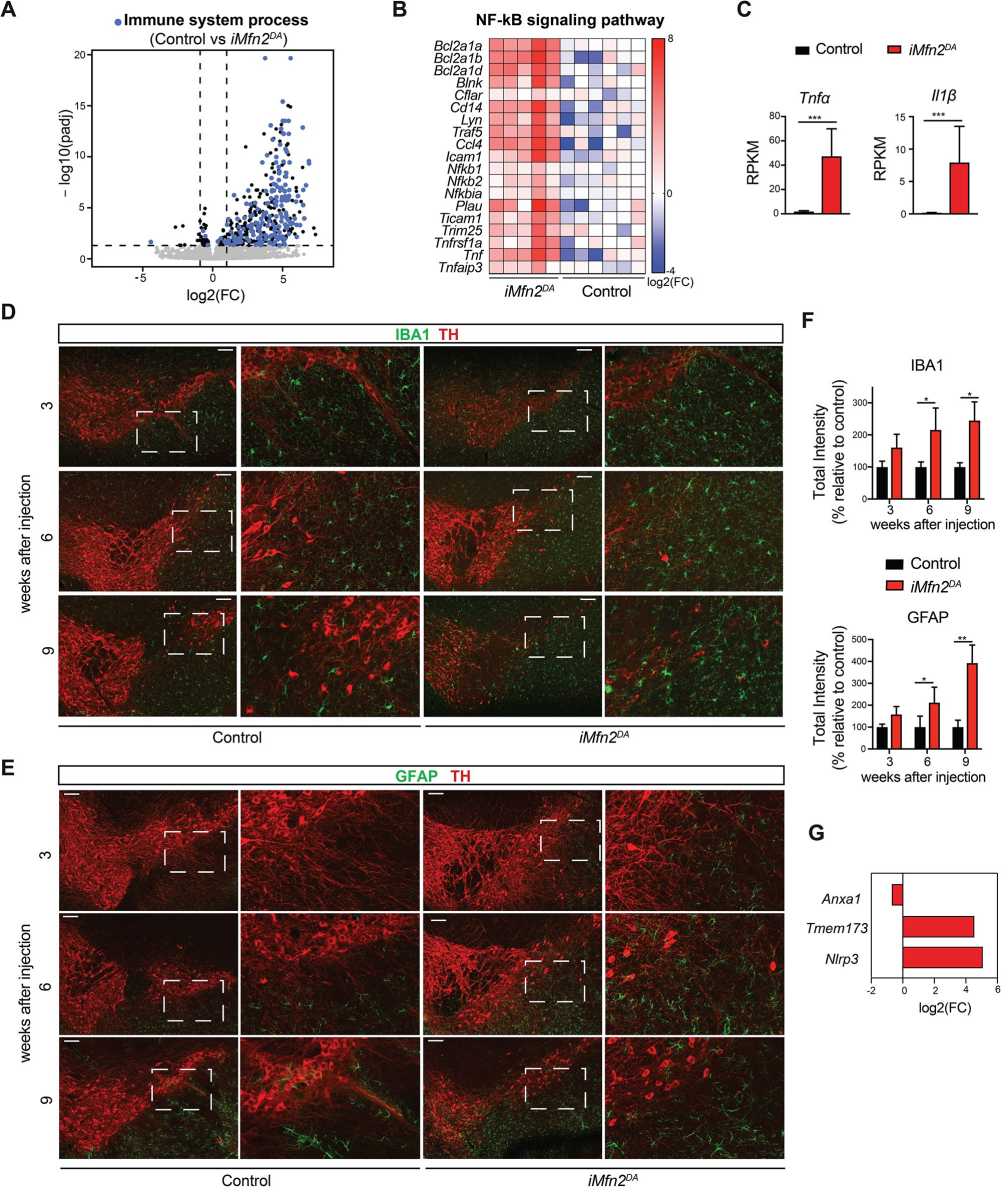
Activation of glial cells surrounding DA neurons in in tamoxifen-injected *iMfn2*^DA^ mice. (A) Volcano plot displaying differential gene expression in *iMfn2*^DA^ FACS-sorted DA neurons: the 439 differentially expressed genes are represented in black and blue. The genes involved in the immune response are represented in blue. (B) Heatmap showing the gene expression of NF-kB signaling pathway in DA neurons isolated from *iMfn2*^DA^ and control mice. (C) RNA expression levels (RPKM) of the inflammation markers *Tnf-α* and *IL1β* in mitoYFP+ cells from *iMfn2*^DA^ and control mice. Data are shown as mean ± SD. (D-E). Representative confocal microscopy image of control and *iMfn2*^DA^ mouse brains at 3, 6, and 9 weeks after injection. DA neurons were labelled with an antibody against TH (red) and the brain sections were additionally labelled with antibody against IBA1 (green) in panel (D) or GFAP (green) in panel (E) (Scale bars: 100 μm). (F) IBA1 and GFAP immunoreactivities quantified as total intensity in the stained areas of the midbrain from control and tamoxifen-injected *iMfn2*^*DA*^ mice at 3, 6 and 9 weeks after injection. Data are shown as mean ± SD. n≥3.*p< 0.05, **p<0.01. (G) Log2(FC) of RNA expression of genes involved in signaling pathways that potentially could drive neuroinflammation in *iMfn2*^DA^ mice.

To gain further insights into the molecular mechanisms that precede and contribute to loss of DA neurons, we compared the transcriptome profiles of FACS-sorted *iMfn2*^DA^ and control DA neurons at 3 weeks after tamoxifen injection. By using *DESeq2*, 439 genes were found differentially expressed at adjusted p value (padj) of <0.05 (listed in S1 Table). Gene ontology and pathway enrichment analyses were performed to identify functional categories of these genes. Unexpectedly, at this early-disease stage, when OXPHOS function, mtDNA levels and DA neuronal survival were unaffected, the molecular pathways related to immune response and inflammation were the most dysregulated biological processes in tamoxifen-injected *iMfn2*^DA^ mice (Fig 4E). The vast majority of significantly upregulated genes belonged to immune system processes (Fig 5A), whose activation was mediated by the NF-KB pathway (Fig 5B). Furthermore, the expression levels of pro-inflammatory cytokines, such as tumor necrosis factor α (Tnf-α) and interleukin-1 β (IL-1β) were dramatically increased in tamoxifen-injected *iMfn2*^DA^ mice (Fig 5C). Control mice injected with tamoxifen showed no activation of the immune response (Fig 5B and 5C).

### Adult-onset mitochondrial dysfunction in DA neurons triggers the activation of surrounding glial cells

To further investigate the immune response observed in tamoxifen-injected *iMfn2*^DA^ mice, we analyzed the glial cells, microglia and astrocytes, surrounding DA neurons by confocal microscopy. At 3 weeks after tamoxifen injection, the immunoreactivities of IBA1 and CD45, markers of activated microglia, were moderately increased (~1.6 fold) in midbrain sections of *iMfn2*^DA^ mice (Figs 5D, 5F, S3B and S3C). Consistently, the transcript levels of different markers of reactive microglial cells, including *Aif1* (*Iba1*), *Tmem119*, *Ptprc* (*Cd45)*, and *Itgam* (*Cd11b*), were significantly upregulated in *iMfn2*^DA^ mice (S3D Fig). Likewise, the levels of the glial fibrillary acidic protein (GFAP) were ~1.5 fold higher in the astrocytes residing in the midbrain and surrounding the DA neurons. (Fig 5E and 5F). Between 6 and 9 weeks after tamoxifen injection there was only a mild upregulation of IBA1 and CD45 (~1.8–2 fold), whereas GFAP signal markedly accumulated over time (up to ~4 fold) (Figs 5D–5F, S3B and S3C) suggesting that at 9 weeks after injection astrocytes were strongly activated, which closely resembled the reactive astrogliosis observed in the late stages of neurodegenerative diseases [25].

Finally, to identify genes potentially involved in the signaling between neurons, microglia, and astrocytes, we interrogated the transcriptomic data. Importantly, DA neurons lacking *Mfn2* showed a significant downregulation of *Anxa1* (Fig 5F) encoding the anti-inflammatory mediator Annexin A1 (ANXA1) [26], which is normally highly expressed in DA neurons [27]. The reduction in *Anxa1* gene expression in tamoxifen-injected *iMfn2*^DA^ mice can potentially explain the upregulation of pro-inflammatory markers. In support of this hypothesis, it has been previously shown that *Anxa1* overexpression in neuronal cells treated with the complex I inhibitor methyl-4-phenylpyridinium (MPP+) can suppress pro-inflammatory responses [28]. Furthermore, the expression of both *Tmem173* (STING) and *Nlrp3* inflammasome genes were significantly increased in tamoxifen-treated *iMfn2*^DA^ mice (Fig 5F). Along the same lines, activation of the NLRP3 or STING pathways [29], triggered by danger-associated molecular patterns (DAMPs) originating from mitochondria [30], causes a detrimental immune response in mice lacking mitochondrial transcription factor TFAM in DA neurons [31] and in *Parkin* and *Pink1* knockout mice after exhaustive exercise [32].

To summarize, we report here that adult-onset mitochondrial dysfunction in DA neurons leads to degeneration of these neurons, DA depletion in the striatum and reduction of voluntary movement. Mice lacking *Mfn2* in the mature nigrostriatal system represent a novel model that well recapitulates major pathological features of human PD. Our results provide compelling evidence that mitochondrial integrity, preserved through an intact mitochondrial fusion machinery, is not only required during embryonic development but it is also essential for the maintenance of the adult DA neuron population. To dissect the timeline of the molecular events leading to neurodegeneration, we exploited bulk RNA-seq of isolated midbrain DA neurons at an early-disease stage. Our protocol resulted in a very substantial enrichment of midbrain DA neurons in both controls and knockouts (Fig 4C), although a minor contamination of other cell types, e.g. neighboring glial cells, was likely present in our samples (S3E Fig). Microglial markers were indeed significantly increased in the mitoYFP positive cells isolated from tamoxifen-injected *iMfn2*^DA^ mice (S3E Fig), as these genes become highly expressed upon glial activation. It is therefore possible that the changes in transcriptomic profile of *iMfn2*^DA^ samples were partially affected by a concomitant response in glial cells surrounding DA neurons. Nevertheless, the conclusions of this study do not change as, in fact, our results highlight that the adult-onset loss of mitochondrial homeostasis triggers an early immune response that largely precedes DA neuron death and likely promotes or exacerbates the degenerative process. Interestingly, recent studies report that *Mfn2* ablation in the adult mouse hippocampus and neocortex causes neuronal cell death through neuroinflammation [33,34]. The progression of these molecular defects resembles the order of pathological events that we have observed in the *Substantia nigra* in the absence of *Mfn2* suggesting that inflammation may be a common early event in degeneration of different neuronal types, e.g. pyramidal, cortical and DA neurons.

Numerous studies have shown that neuroinflammation is a major player in PD and may contribute to the degeneration of the nigrostriatal DA pathway, promoting disease progression [35]. Post-mortem examinations have revealed large numbers of reactive microglial cells [36] and high levels of pro-inflammatory modulators in the brain [37,38] and biological fluids [39,40] of PD patients. Nevertheless, it has remained unclear whether neuroinflammation is merely a downstream effect of nerve cell death [41] or if it is primarily involved in PD pathogenesis. The *in vivo* data presented here corroborate the hypothesis that DA neuron loss in the adult brain is strongly facilitated by early onset of neuroinflammation, which supports the importance of non-cell autonomous mechanisms in the neurodegenerative process. Based on these findings, we propose that fully differentiated DA neurons can generate a signal that activates glial cells in response to defective mitochondrial function. The release of a multitude of immunomodulatory molecules, including pro-inflammatory modulators, likely has a cytotoxic effect inducing damage to neighboring neurons. Eventually, a self-propelling vicious cycle may ensue driving a continuously ongoing degeneration of DA neurons. Further studies are required to define details of the molecular mechanisms linking defective mitochondrial function to altered immune response. The relevance of these findings for the pathophysiology of PD needs verification by studies of human tissues.

## Materials and methods

### Ethics statement

All animal procedures were conducted in accordance with European, national and institutional guidelines and protocols were approved by the Stockholm ethical committee (Stockholms djurförsöksetiska nämnd) under the ethical permit 1206–2019. Animal work also followed the guidelines of the Federation of European Laboratory Animal Science Associations (FELASA).

### Mouse models

Mice homozygous for a loxP-flanked *Mfn2* allele (*Mfn2 loxP/loxP*) [20] were crossed to heterozygous *DATcreERT2* mice [42]. Double heterozygous offspring was obtained and crossed with *Mfn2 loxP/loxP* mice to generate *iMfn2*^DA^ and control mice. The Gt(ROSA26)Sor^Stop–mito–YFP^ allele (stop-mitoYFP) [13], that when activated express mitochondrially targeted YFP, was subsequently introduced via additional crossing. At 5–7 weeks of age, *iMfn2*^DA^ mice were treated for 5 consecutive days by intraperitoneal injection of 2 mg of tamoxifen (Sigma T5648 dissolved in ethanol and sunflower oil) or vehicle. Two different control groups were employed: the first group (*Mfn2 loxP/loxP*; *DATcreERT2/+*) was injected with vehicle and used to assess motor performance and survival, the second control group (*Mfn2 wt/wt; stop-mitoYFP/wt; DATcreERT2/wt*) was injected with tamoxifen and used to visualize mitochondria and isolate DA neurons from animals with normal mitochondrial function. Analyses of injected controls and KO mice were performed at 3, 6, and 9 weeks after the last injection. All mice were on the C57BL/6N background.

### Motor performance

The motor activity of vehicle injected control (n>14) and tamoxifen injected *iMfn2*^DA^ (n>14) mice was measured by an open field test (VersaMax, AccuScan Instruments) at 3, 6, and 9 weeks after injection. Following an acclimation period of at least 30 min in the ventilated experimental room, mice were placed individually in activity cages (40 × 40 cm and 30 cm high) for 60 minutes during the same period (between 4–6 p.m.). A grid of infrared light beams at floor level and 7.5 cm above recorded spontaneous horizontal and vertical activities and the total distance travelled was calculated.

### *In situ* hybridization

The expression of *Th* and *Mfn2* transcripts in the DA neurons of the SN was detected as previously described [20].

### Western blot

Ventral midbrain was dissected from control and tamoxifen-injected *iMfn2*^*DA*^ mice and snap frozen in liquid nitrogen. Tissue was homogenized in RIPA buffer supplemented with protease inhibitors (Complete, Roche). Twenty micrograms of protein extracts were resuspended in Laemmli buffer, run on 12% SDS–polyacrylamide gel electrophoresis (Invitrogen) and then transferred onto polyvinylidene difluoride membranes (GE Healthcare). Blots were incubated overnight at 4°C with primary antibody against MFN2 (ab 56889, Abcam) and GAPDH (ab8245, Abcam). Immunodetection was performed according to standard techniques using enhanced chemiluminescence Immun-Star HRP Luminol/Enhancer (Bio-Rad).

### Immunohistochemistry and confocal microscopy

Mice were perfused with Ca^2+^/Mg^2+^ free Tyrode’s solution followed by 4% paraformaldehyde with 0.4% picric acid in 0.16 M phosphate buffer. The brains were dissected, postfixed for 2 hours, and equilibrated with 10% sucrose. Brains were frozen and cryo-sectioned to obtain 14 (for the striatum) or 20 (for the midbrain) μm thick sections. After 1 hour in blocking solution (PBS+ 0.3% TrItox X-100+ 1% BSA), the tissue sections were immunolabelled overnight with primary antibodies against TH (1:500, Pel-Freez; 1:1000, Chemicon), IBA1 (1:1000, Wako), GFAP (1:1000, Abcam) and CD45 (1:100, Serotec). For fluorescent staining, Cy3- (1:400, Jackson Biolabs), Alexa 546- (1:400, Life Technologies) and Alexa 633- conjugated secondary antibodies (1:400, Life Technologies) were used. Confocal images were acquired by sequential scanning using a LSM800 or LSM880 microscope (Zeiss). Relative intensity of mitoYFP-labelled mitochondria in the striatum and IBA1, CD45 and GFAP immunoreactivities in the midbrain were quantified using Fiji software. Confocal images were thresholded and total intensity was measured using automatic particle counting.

Live microscopy on sorted cells was performed as previously described [43].

### Quantification of TH+ neurons and nerve terminals

Vehicle (n = 3) and tamoxifen-injected (n = 3) *iMfn2*^DA^ mice were perfused at 3, 6 and 9 weeks after injection and the brains were cryo-sectioned. Every sixth midbrain cryo-section (20 μm thickness) was immunolabelled for TH. For the non-fluorescent labelling, a biotinylated secondary antibody (1:400, Vector Laboratories) was used and the signal was detected by using a peroxidase substrate (Vector SG, Vector Laboratories). Nuclei of TH-positive neurons were counted in both right and left hemisphere from 9–11 sections for brain. For the quantification of nerve terminals in the striatum, the sections were immunolabelled with antibodies against TH and fluorescent secondary antibodies. Fiji software was used for the measurement of TH density.

### Quantification of mitochondrial morphology

Mitochondrial morphology analysis of confocal pictures was performed using Fiji software. TH-positive cells were used to outline the area of interest and apply to the mitochondrial channel. The mitochondria were measured using a macro containing the Fiji default shape descriptors after applying the same threshold to controls and KO animals. Aspect ratio (AR) of each mitochondrial object was expressed as the ratio of the major axis/minor axis. The circularity was calculated as 4π *area/perimeter^2. Results represent a minimum of 12 cells from n = 3 mice per condition (1 = perfectly rounded object; 0 = elongated object).

### Dual COX/SDH enzyme histochemistry

Vehicle (n = 3) and tamoxifen-injected (n = 3) *iMfn2*^DA^ mice were euthanized with carbon dioxide and decapitated, Brains were rapidly collected and frozen on dry ice. Brain sections (14 μm) were stained as previously described [44].

### Measurements of neurotransmitters

Brains from tamoxifen-injected control and *iMfn2*^DA^ mice (n≥ 5 per genotype) were rapidly dissected, chilled in ice-cold saline and bilateral striatal pieces of the striatum were frozen on dry ice. Metabolites were extracted from 10–20 mg of mouse tissues in ice cold extraction buffer (0.37% formic acid in water). After homogenization, the samples were pelleted by centrifugation (21000 x g) for 5 minutes at 4°C. The obtained supernatant was de-proteinated by centrifugation at 4°C for 10 minutes through a 5 kDa cut-off size exclusion filter (Microcons, Millipore). The flow-through containing the acidified metabolic extracts (DA, HVA and 5-HT) was immediately separated by HPLC on a reversed phase UPLC column (100 mm x 2.1 mm, C18, Hypersil Gold) held at 25°C. The eluting metabolites were detected in positive ionization mode using a ESI MRM (ElectroSpray Ionization Multi Reaction Monitoring) method. Absolute quantification of the analyzed compounds was obtained by converting the obtained peak areas from the samples into concentrations derived from the analysis of calibration curves. Data analysis and peak integration were performed using the TargetLynx software.

### Electron microscopy

The mice (n = 3 for each genotype and condition) were perfused with 4% PFA and 0.1% glutaraldehyde (Merck) in PBS at 3, 6, and 9 weeks after tamoxifen injection. Brains were dissected and postfixed for 4 hours at 4°C in the same fixative, washed in PBS, and cut into 100 μm slices with a vibratome (Leica, Germany). Free-floating sections were blocked in 0.1% Triton X-100 and 10% donkey serum in PBS for 1 hour at room temperature, incubated with primary rabbit anti-TH antibodies (1:1000, Pel-Freeze) in PBS for 8 hours and secondary donkey anti-rabbit antibodies conjugated to biotin (1:200, Jackson ImmunoResearch Laboratories) for 4 hours, and stained using Vectastain ABC and DAB kits (Vector Laboratories). The sections were postfixed in 3% glutaraldehyde and 1% osmium tetroxide, dehydrated in ethanol and embedded in Durcupan ACM resin (Fluka). In several experiments, sections were stained with 1% uranyl acetate in 70% ethanol. Serial ultrathin (70 or 100 nm) and semithin (1 μm) sections were cut with diamond knives (Diatome). Ultrathin sections were collected onto formvar-coated copper grids, counterstained with 1% uranyl acetate and lead citrate and examined in a Tecnai 12 electron microscope (FEI) equipped with a 2kx2k TemCam-F224HD camera (TVIPS). Complete series of up to 150 ultrathin sections were used to follow the morphology of cells and synaptic terminals in three dimensions. The mitochondrial aspect ratio and length were quantified in two TH positive cells for each genotype at 3 weeks after injection. For each cell, the morphology of 100 mitochondria was analyzed using 3D reconstructed images from serial ultrathin sections. Quantification of the relative mitochondrial mass (mitochondrial area/cytosol) in assessed in TH positive neurons (n>16 cells) for each genotype and time point.

### Isolation of DA neurons by FACS

Brains were dissected from mitoYFP expressing tamoxifen-injected control and *iMfn2*^DA^ mice, sectioned and dissociated into single cell suspensions, as previously described [45]. After dissociation, the mitoYFP positive cells were isolated using a BD FACSAria III Cell Sorter and collected for mtDNA quantification or RNAseq.

### mtDNA measurement

Bulks of 100 mitoYFP positive neurons (n = 4 for genotype and time point) were collected in lysis buffer (50 mM Tris-HCl pH 8.5, 1 mM EDTA, 0.5% Tween-20, 200 ng/mL Proteinase K). After centrifugation (7000 g for 10 minutes), DNA extraction was performed at 55°C for 2 hours followed by 10 minutes at 95°C to denature Proteinase K. Quantification of mtDNA copy number was performed using TaqMan Universal Master Mix II and TaqMan probes against the mitochondrial genes (ND1 and ND6) from Life Technologies. The nuclear 18S rRNA gene was used as an internal standard.

### Library preparation and sequencing

FACS sorted cells (n≥5 per genotype) were used to generate the cDNA libraries according to the Smartseq2 protocol [23] as previously described [46]. The Nextera XT DNA library preparation kit (FC-131-1024) was used for cDNA tagmentation. The quality of cDNA and tagmented cDNA was checked on a High-Sensitivity DNA chip (Agilent Bioanalyzer). Sequencing was performed on Illumina HiSeq 2500, giving 51 bp reads after de-multiplexing.

### Read alignment and gene expression analysis

Reads were aligned to the mouse genome (mm10) merged with eGFP and ERCC spike-in sequences using Star v2.3.0 [47] and filtered for uniquely mapping reads. Gene expression was calculated as read counts and as reads per kilobase gene model and million mappable reads (RPKMs) for each transcript in *Ensembl release 75* using rpkmforgenes [48]. Experiments were performed in technical replicates that were merged. Read counts were summed across technical replicates and RPKMs were averaged across technical replicates, resulting in gene expression data for 6 control and 5 *iMfn2*^DA^ mice. The 23217 protein-coding genes based on the gene and transcript classification in *Ensembl release 75* were selected for further analyses.

For hierarchical clustering, gene counts were first VST-transformed [49], and then the 10% most varying protein-coding genes (n = 2322) were selected and mean-centered across the 11 biological replicates (6 controls and 5 *iMfn2*^DA^ mice). Hierarchical clustering was performed in R using the ward.D2 agglomerative method and the Pearson correlation-based distance measure. Differential gene expression analysis was performed using DESeq2 [50]. Gene set enrichment analysis was performed using DAVID Bioinformatics Resources 6.8.

### Statistical analysis

All statistical analyses were performed using GraphPad Prism v6 software. All data in the figures are presented as mean ± SD. Statistical comparisons were performed using single or multiple Student’s t-test or one-way analysis of variance (ANOVA).

## Supporting information

S1 Fig Loss of *Mfn2* in adult DA neurons results in a lethal phenotype. (A) Diagram depicting tamoxifen-induced inactivation of the *Mfn2* gene in adult *iMfn2*^DA^ mice. Mice at 5–7 weeks of age were intraperitoneally injected with tamoxifen for 5 consecutive days and examined for 3–9 weeks after injection. (B) *In situ* hybridization showing the expression of *Mfn2* and *Th* transcripts in DA neurons of SN (in the red box). (C) Western blot analysis of MFN2 protein levels in total extracts from ventral midbrain of control and knockout mice at 3 weeks after tamoxifen injection. GAPDH was used as a loading control. (D) Survival of *iMfn2*^DA^ and control mice after tamoxifen injection. *iMfn2*^DA^ mice had a median survival of 11,6 weeks. ***p < 0.001 n = 15. (E) Body weight of control and *iMfn2*^DA^ mice (males and females) after tamoxifen or vehicle injection. **p < 0.01 n>10. (F) Analysis of 5-HT levels in the striatum at 3, 6, and 9 weeks after tamoxifen injection. n≥5. Data are shown as mean ± SD.
(TIF)

S2 Fig Mitochondrial morphology in tamoxifen-injected *iMfn2*^DA^ mice. (A) Visualization of mitochondria in DA neurons *in vivo*. The expression of mitoYFP (green) overlaps with TH (red) labelling of midbrain DA neurons (Scale bars: 50 μm). (B) Representative confocal microscopy images of mitoYFP-labelled mitochondria (green) in TH immunoreactive neurons (red) at 2 weeks after tamoxifen injection (Scale bar: 10 μm). (C) Representative transmission electron microscopy images of DA neurons. The lines mark the nuclear (N) and the plasma membrane (Scale bars: 5 μm). (D) Quantification of aspect ratio and mitochondrial length at 3 weeks after injection in two cells for each genotype using serial ultrathin sections. ***p< 0.001. (E) Quantification of the relative mitochondrial mass (mitochondrial area/cytosol) in TH+ DA neurons from EM images at 3, 6 and 9 weeks after injection. ***p< 0.001, n>16 cells for each genotype. (F) EM images of mitochondria from perinuclear region of DA neurons with disrupted OMM 6 and 9 weeks after tamoxifen injection (Scale bar: 1μm). (G) Electron micrographs of DA nerve terminals, delineated by the light blue lines, in striatum 6 and 9 weeks after tamoxifen injection (Scale bars: 500 nm).
(TIF)

S3 Fig Immune response in tamoxifen-injected *iMfn2*^DA^ mice. (A) Representative confocal microscopy images of mitoYFP+ cells obtained with the DA neuron isolation protocol from control mice injected with tamoxifen (Scale bar: 5 μm and 20 μm). (B) Representative confocal microscopy images of control and *iMfn2*^DA^ mouse midbrain 3, 6, and 9 weeks after tamoxifen injection. The brain sections were stained with antibodies against CD45 (red) and TH (blue) (Scale bars: 50 μm). (C) CD45 immunoreactivity quantified as total intensity in the stained areas of the midbrain from control and tamoxifen-injected *iMfn2*^*DA*^ mice at 3, 6 and 9 weeks after injection. Data are shown as mean ± SD. n≥3.*p< 0.05. (D) RNA expression levels (RPKM) of the markers of activated microglial cells *Aif1* (Iba1), *Tmem119*, *Ptprc* (*Cd45)* and *Itgam* (Cd11b) in mitoYFP+ samples isolated from *iMfn2*^DA^ and control mice. (E) Cell-type markers in isolated mitoYFP+ and mitoYFP- cells. Heatmaps showing RNA levels (RPKM) of genes encoding: ii) microglial and ii) astrocyte markers, which are more abundant in mitoYFP- samples. Data are shown as mean of the RPKM in control (n = 6), *iMfn2*^*DA*^ (n = 5), and mitoYFP- (n = 3) mice.
(TIF)

S1 Table List of the 439 genes found differentially expressed in *iMfn2*^DA^ mice at adjusted p value (padj) of <0.05. (XLSX)
