# Supplementary figures and images for: Mitochondrial dysfunction in adult midbrain dopamine neurons triggers an early immune response
Source: PLoS Genet. 2021 Sep 27;17(9):e1009822. doi: 10.1371/journal.pgen.1009822 (PMC8496783; doi:10.1371/journal.pgen.1009822)

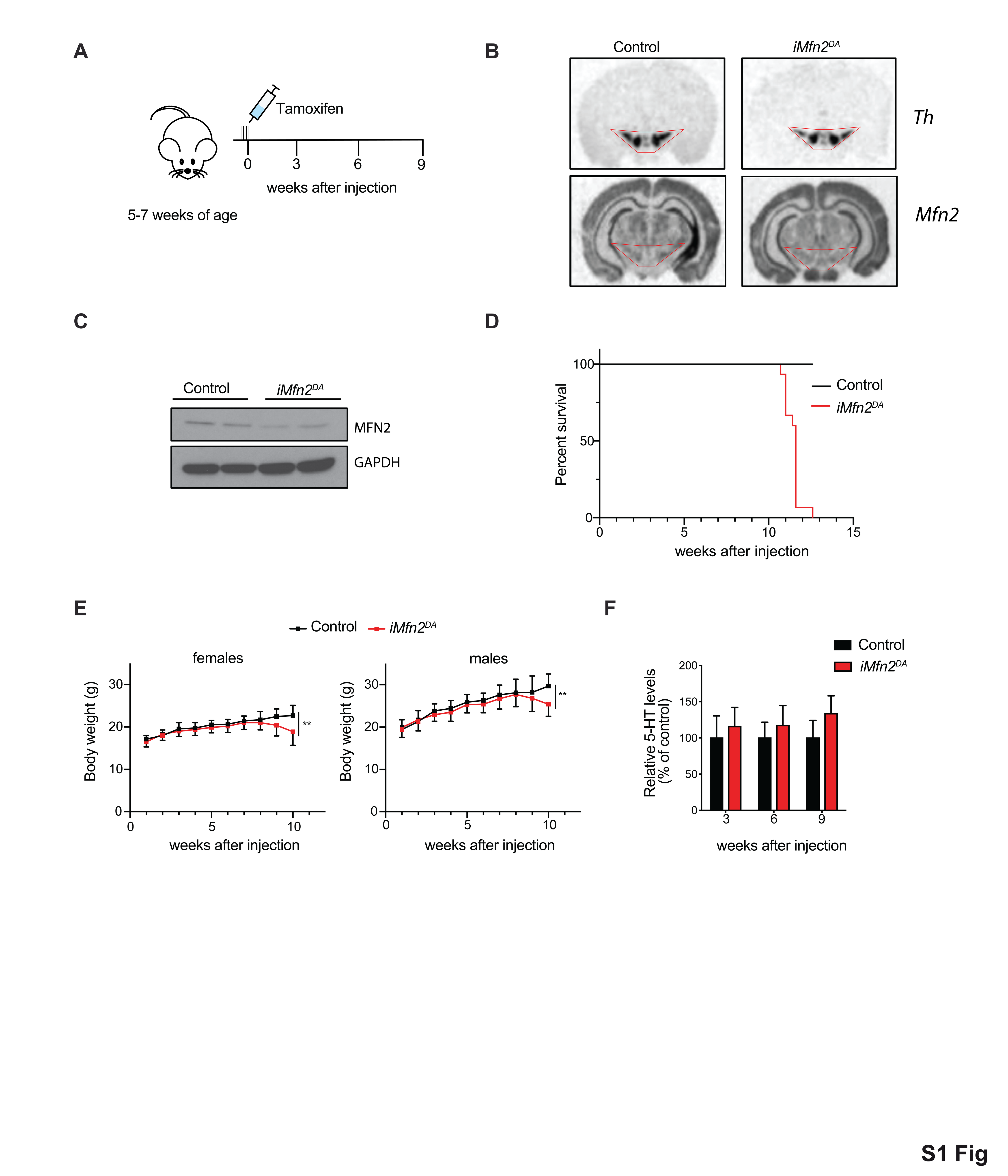

Supplement: S1 Fig — (A) Diagram depicting tamoxifen-induced inactivation of the Mfn2 gene in adult iMfn2DA mice. Mice at 5–7 weeks of age were intraperitoneally injected with tamoxifen for 5 consecutive days and examined for 3–9 weeks after injection. (B) In situ hybridization showing the expression of Mfn2 and Th transcripts in DA neurons of SN (in the red box). (C) Western blot analysis of MFN2 protein levels in total extracts from ventral midbrain of control and knockout mice at 3 weeks after tamoxifen injection. GAPDH was used as a loading control. (D) Survival of iMfn2DA and control mice after tamoxifen injection. iMfn2DA mice had a median survival of 11,6 weeks. ***p < 0.001 n = 15. (E) Body weight of control and iMfn2DA mice (males and females) after tamoxifen or vehicle injection. **p < 0.01 n>10. (F) Analysis of 5-HT levels in the striatum at 3, 6, and 9 weeks after tamoxifen injection. n≥5. Data are shown as mean ± SD. (TIF) [file pgen.1009822.s001.tif]

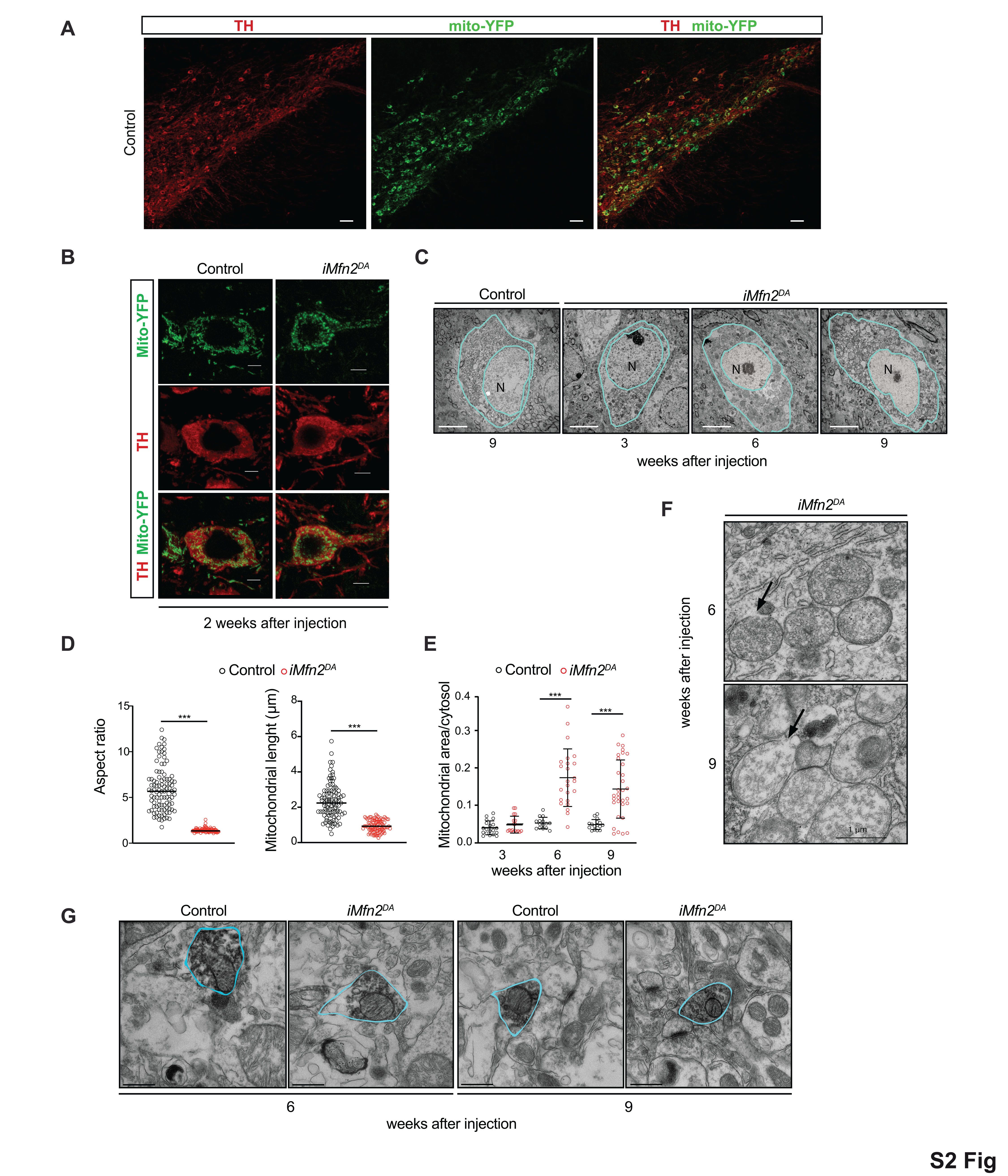

Supplement: S2 Fig — (A) Visualization of mitochondria in DA neurons in vivo. The expression of mitoYFP (green) overlaps with TH (red) labelling of midbrain DA neurons (Scale bars: 50 μm). (B) Representative confocal microscopy images of mitoYFP-labelled mitochondria (green) in TH immunoreactive neurons (red) at 2 weeks after tamoxifen injection (Scale bar: 10 μm). (C) Representative transmission electron microscopy images of DA neurons. The lines mark the nuclear (N) and the plasma membrane (Scale bars: 5 μm). (D) Quantification of aspect ratio and mitochondrial length at 3 weeks after injection in two cells for each genotype using serial ultrathin sections. ***p< 0.001. (E) Quantification of the relative mitochondrial mass (mitochondrial area/cytosol) in TH+ DA neurons from EM images at 3, 6 and 9 weeks after injection. ***p< 0.001, n>16 cells for each genotype. (F) EM images of mitochondria from perinuclear region of DA neurons with disrupted OMM 6 and 9 weeks after tamoxifen injection (Scale bar: 1μm). (G) Electron micrographs of DA nerve terminals, delineated by the light blue lines, in striatum 6 and 9 weeks after tamoxifen injection (Scale bars: 500 nm). (TIF) [file pgen.1009822.s002.tif]

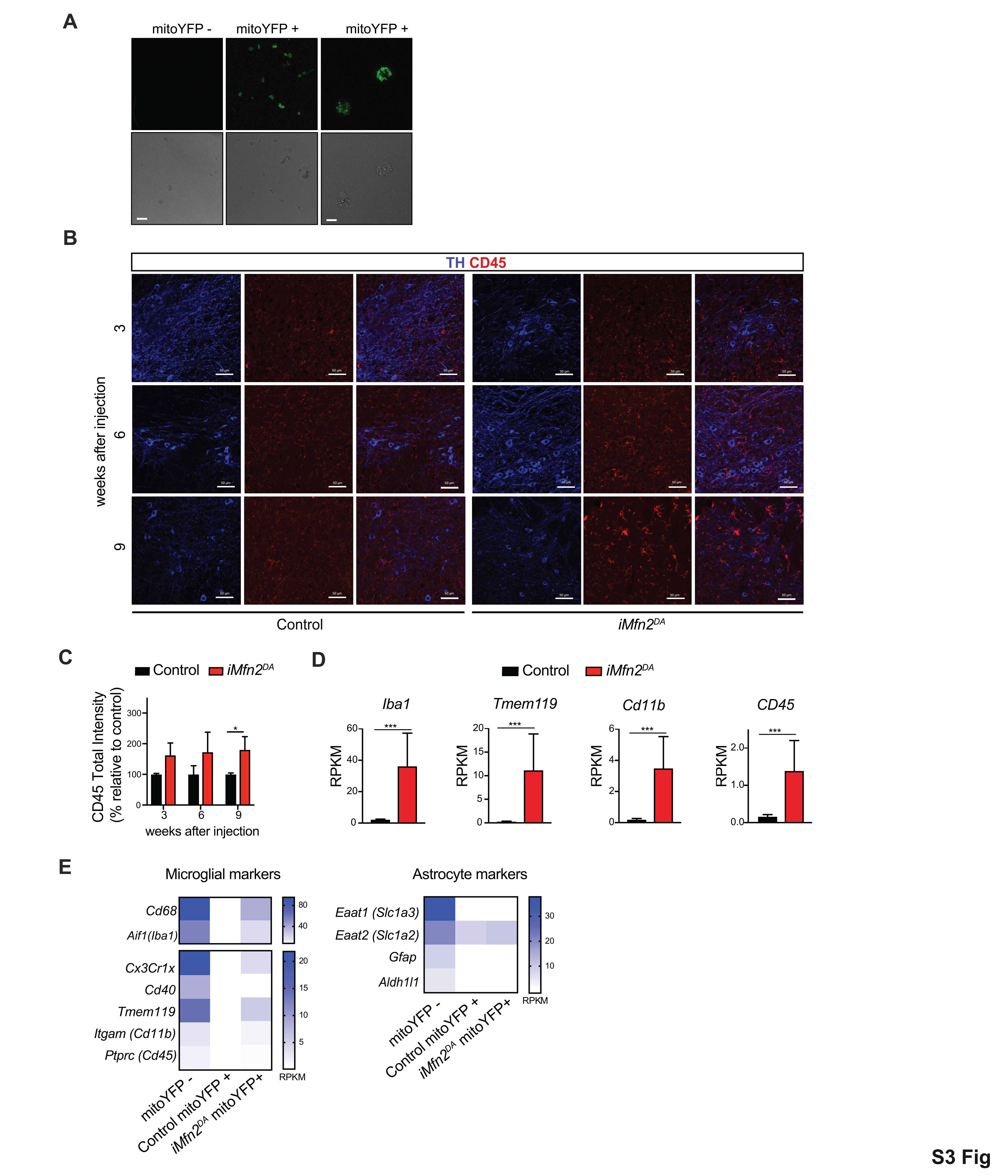

Supplement: S3 Fig — (A) Representative confocal microscopy images of mitoYFP+ cells obtained with the DA neuron isolation protocol from control mice injected with tamoxifen (Scale bar: 5 μm and 20 μm). (B) Representative confocal microscopy images of control and iMfn2DA mouse midbrain 3, 6, and 9 weeks after tamoxifen injection. The brain sections were stained with antibodies against CD45 (red) and TH (blue) (Scale bars: 50 μm). (C) CD45 immunoreactivity quantified as total intensity in the stained areas of the midbrain from control and tamoxifen-injected iMfn2DA mice at 3, 6 and 9 weeks after injection. Data are shown as mean ± SD. n≥3.*p< 0.05. (D) RNA expression levels (RPKM) of the markers of activated microglial cells Aif1 (Iba1), Tmem119, Ptprc (Cd45) and Itgam (Cd11b) in mitoYFP+ samples isolated from iMfn2DA and control mice. (E) Cell-type markers in isolated mitoYFP+ and mitoYFP- cells. Heatmaps showing RNA levels (RPKM) of genes encoding: ii) microglial and ii) astrocyte markers, which are more abundant in mitoYFP- samples. Data are shown as mean of the RPKM in control (n = 6), iMfn2DA (n = 5), and mitoYFP- (n = 3) mice. (TIF) [file pgen.1009822.s003.tif]
